# Hypoxia induces differential translation of enolase/MBP-1

**DOI:** 10.1186/1471-2407-10-157

**Published:** 2010-04-22

**Authors:** Kara C Sedoris, Shelia D Thomas, Donald M Miller

**Affiliations:** 1 James Graham Brown Cancer Center, Department of Medicine, University of Louisville School of Medicine, Louisville, Kentucky 40202, USA

## Abstract

**Background:**

Hypoxic microenvironments in tumors contribute to transformation, which may alter metabolism, growth, and therapeutic responsiveness. The α-enolase gene encodes both a glycolytic enzyme (α-enolase) and a DNA-binding tumor suppressor protein, c-myc binding protein (MBP-1). These divergent α-enolase gene products play central roles in glucose metabolism and growth regulation and their differential regulation may be critical for tumor adaptation to hypoxia. We have previously shown that MBP-1 and its binding to the c-myc P_2 _promoter regulates the metabolic and cellular growth changes that occur in response to altered exogenous glucose concentrations.

**Results:**

To examine the regulation of α-enolase and MBP-1 by a hypoxic microenvironment in breast cancer, MCF-7 cells were grown in low, physiologic, or high glucose under 1% oxygen. Our results demonstrate that adaptation to hypoxia involves attenuation of MBP-1 translation and loss of MBP-1-mediated regulation of c-myc transcription, evidenced by decreased MBP-1 binding to the c-myc P_2 _promoter. This allows for a robust increase in c-myc expression, "early c-myc response", which stimulates aerobic glycolysis resulting in tumor acclimation to oxidative stress. Increased α-enolase mRNA and preferential translation/post-translational modification may also allow for acclimatization to low oxygen, particularly under low glucose concentrations.

**Conclusions:**

These results demonstrate that malignant cells adapt to hypoxia by modulating α-enolase/MBP-1 levels and suggest a mechanism for tumor cell induction of the hyperglycolytic state. This important "feedback" mechanism may help transformed cells to escape the apoptotic cascade, allowing for survival during limited glucose and oxygen availability.

## Background

It is clear that the tumor microenvironment influences the rate of cell proliferation and may have a profound effect on tumor progression and resistance to therapy [1,2]. As a result of variable blood flow (oxygen supply) and rapid utilization of glucose within solid tumors (oxygen utilization), most tumor cells are subjected to a microenvironment that is hypoxic and may be hypoglycemic. These conditions likely contribute to tumor transformation and growth [3,4]. Regional tumor hypoxia develops in the early stages of carcinogenesis before tumor metastasis, even non-invasive tumors may be hypoxic [5,6]. Hypoxia is quite common in breast cancer where it has been related to poor prognosis [7] with increased risk for tumor recurrence and metastasis [8].

Transformed cells demonstrate increased levels of glycolysis, which are associated with increased levels of glycolytic enzyme mRNA and protein [59]. This results in the production of, large amounts of lactic acid (Warburg Effect) [10,11]. This increase in glycolytic metabolism, mediated by Hif-1α and c-myc [12], provides transformed cells with a selective growth advantage by circumventing the normal oxygen dependency for ATP production. Although the changes associated with increased glycolytic enzyme mRNA and protein levels [5,9] have been well documented, the exact mechanisms leading to increased glycolysis and abnormal tumor cell growth under hypoxic conditions are not completely understood.

Because of its frequent overexpression in transformed cells, stimulatory effect on cell growth [13], and ability to upregulate the transcription of several glycolytic enzymes [14] the "early response" gene, c-myc, has been implicated in adaptation of transformed cells to hypoxia [15]. C-Myc is known to be overexpressed in approximately 70% of all human tumors [12]. One of the enzymes whose expression is upregulated by c-myc is α-enolase (48 KDa), which catalyzes the conversion of 2-phosphoenolpyruvate from 2-phosphoglycerate [16]. *α*-Enolase is also a hypoxic stress protein, which may contribute to hypoxic tolerance of tumors by increasing anaerobic metabolism [17]. Its overexpression in tumors at the RNA and protein level has been associated with progression of tumors and poor patient survival [18,19].

Interestingly, α-enolase mRNA also gives rise to a shorter (37 KDa) alternative translation product, c-myc binding protein (MBP-1). In contrast to α-enolase, MBP-1 is a DNA binding protein and does not have enolase activity. MBP-1 is preferentially localized in the cell nucleus and negatively regulates c-myc transcription by binding to the P_2 _promoter [20-22], the predominant c-myc promoter in normal and transformed cells [23]. Constitutive overexpression of MBP-1 reduces invasiveness and colony formation in breast cancer cells, suppresses tumor formation in nude mice [24], and regresses lung tumor growth [25], indicating that it functions as a tumor suppressor. Thus, the interactions between α-enolase, MBP-1, and c-myc represent an important regulatory intersection between energy metabolism and growth control.

Cell proliferation and induction of the hyperglycolytic state are regulated by levels of MBP-1 expression and its binding to the c-myc P_2 _promoter in response to changes in glucose concentration [26]. However, the differential translation of α-enolase and MBP-1 and its relation to the control of cell growth and metabolism under hypoxia has not been characterized. To examine the regulation of α-enolase and MBP-1 by a hypoxic microenvironment, MCF-7 breast cancer cells were cultured under hypoxic (1% O_2_) growth conditions in low (1 nM), physiological (5 mM), or high glucose (25 mM). The levels of expression of α-enolase, MBP-1, and c-myc were compared to cell proliferation and lactate production. This study provides a new mechanism for the regulation of cell growth and metabolism of transformed cells under hypoxia, demonstrating that induction of α-enolase mRNA, preferential translation of α-enolase over MBP-1, and inhibition of MBP-1 function may all be involved in both promoting survival of MCF-7 cells and stimulating cell growth under substrate limitation.

## Results

### Cell proliferation and Viability

**Figure 1:**
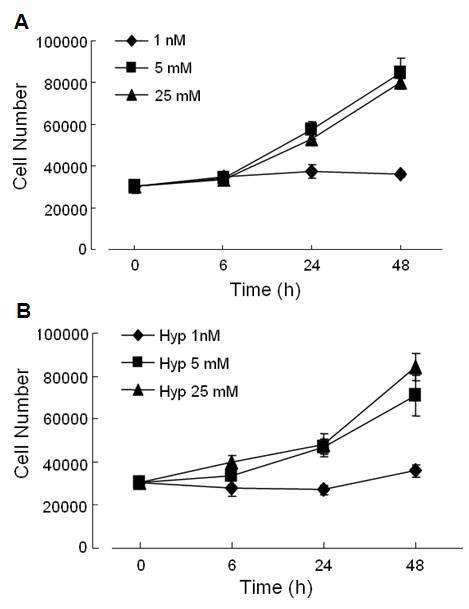
**Effect of hypoxia and different glucose concentrations on cell proliferation**. Changes in cell proliferation after 6, 24, and 48 h of  normoxia (A) or hypoxia (B) in response to 1 nM (diamond), 5 mM (square), or 25 mM (triangle) glucose by trypan blue counting of cells. MCF-7 cells were plated (7.5X10^4^/well) and cell counts and viability were measured. Data is mean ±SEM of 3 separate experiments.

The effect of hypoxia on the proliferation of MCF-7 cells grown in low (1 nM), physiological (5 mM), or high (25 mM) glucose concentrations was determined at 6, 24, and 48 h. Changes in proliferative rate and cellular viability were assessed by trypan blue staining. After 6 h of hypoxia, cell number increased modestly in MCF-7 cells grown in 5 mM and 25 mM glucose and the increase was maintained through 48 h (Fig. 1B). Trypan blue staining demonstrated that the majority of these cells were viable after 48 h of hypoxia (89.0 ± 1.1% and 89.2 ± 2.0% respectively). In contrast, hypoxic cells grown in low glucose grew much more slowly, however, the majority (93.7 ± 2.7%) of the cells remained viable. There was no significant difference in cell number or viability between cells grown in 5 mM or 25 mM glucose under hypoxia or normoxia (Fig. 1A). However, MCF-7 cells grown in low glucose demonstrated decreased proliferative rates under both hypoxic and normoxic conditions.

### Cell Cycle Analysis

Since no change in the rate of proliferation was seen in MCF-7 cells grown under hypoxia at any glucose concentration, we elected to characterize the effect of hypoxia on cell cycle kinetics. Consistent with the data shown above, there was very little effect of hypoxia on the cell cycle distribution in cells grown in normal or high glucose. On the other hand, cells grown in 1 nM glucose demonstrated a significant G_0_/G_1 _arrest (data not shown). This was seen in both normoxic and hypoxic cells, suggesting that hypoxia, per se, does not significantly alter cell cycle distribution. The observation that low glucose inhibits cell proliferation is consistent with previously reported results [26].

### Lactate Production

**Figure 2:**
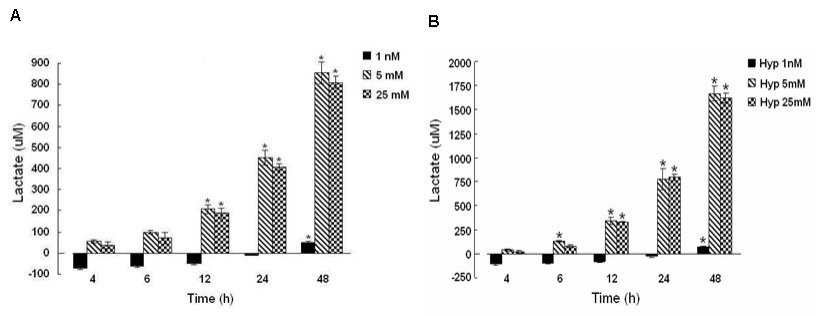
**Lactate production (μM) in medium of MCF-7 cells growing in 1 nM (black bar), 5 mM (striped), or 25 mM (checkered) glucose after 4, 6, 12, 24, or 48 h of normoxia (A) or hypoxia (B)**. Lactate concentrations were determined by detection of NAD-linked conversion of lactate to pyruvate by lactate dehydrogenase at 340 nM. Values are mean ± SEM from 3 separate experiments. * denotes a significant (p < 0.05) increase in lactate compared to baseline. Note a 2-fold increase in lactate production with hypoxia and physiological or high glucose compared to normoxia. Normoxia figure was previously published [26].

In order to determine the effect of hypoxia on the glycolytic rate at each glucose concentration, lactate production by hypoxic cells was assessed over time. Hypoxic MCF-7 cells grown in 1 nM glucose did not produce detectable amounts of lactate from 4-24 h of hypoxia (Fig 2B), however at 48 h, a small amount of lactate was detected. This modest level of lactate production was abrogated when the cells were grown under the same conditions without glutamine (data not shown).

In MCF-7 cells grown in physiologic (5 mM) and high (25 mM) glucose, lactate increased at a linear rate with large increases measured at 12, 24 and 48 h. When compared to MCF-7 cells grown under normoxia [26], hypoxic cells grown in 5 mM and 25 mM glucose produced roughly twice as much lactate from 12-48 h as did normoxic cells (Fig 2A).

### Hypoxia upregulates α-enolase mRNA and protein expression

**Figure 3:**
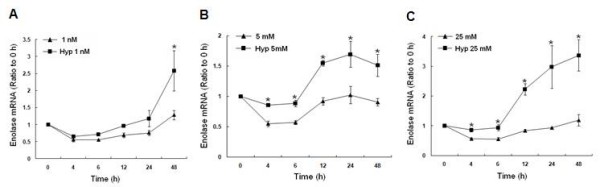
**Time course of a-enolase mRNA after 4, 6, 12, 24, or 48 h of hypoxia in response to 1 nM, 5 mM, or 25 mM glucose by RT-PCR**. All measurements were standardized with β-actin (loading control) and expressed as a ratio to the 0 h (expression ratio). Bars represent mean ± SEM from 3 separate experiments. * denotes a significant (p < 0.05) increase in mRNA compared to normoxia. Note increased α-enolase transcripts after 12-48 h of hypoxia with 1 nM, 5 mM, or 25 mM glucose. Normoxia data was previously published [26].

In order to determine whether α-enolase is upregulated in response to hypoxia, changes in α-enolase mRNA and protein were measured by RT-PCR and Western blot analysis, respectively and compared to normoxic samples. A consistent increase in α-enolase mRNA was seen after 12 to 48 h of hypoxia at all glucose concentrations compared to normoxia. In response to hypoxia and low glucose, α-enolase mRNA levels gradually increased until they were significantly elevated by more than 2.5 fold above normoxia at 48 h (Fig 3). At physiological glucose concentrations, hypoxia immediately increased α-enolase mRNA at 4 h, which continued to rise at 12 h and remain elevated compared to normoxia through 48 h. With 25 mM glucose, hypoxic MCF-7 cells immediately increased α-enolase mRNA after 4 h compared to normoxia. However, after 48 h, α-enolase mRNA increased over 3-fold above normoxia. When compared to MCF-7 cells grown under normoxic conditions [26], hypoxia significantly increased the level of α-enolase mRNA at all glucose concentrations tested. This increase was maximal after 48 h of hypoxia in cells grown in low or high glucose concentrations.

**Figure 4:**
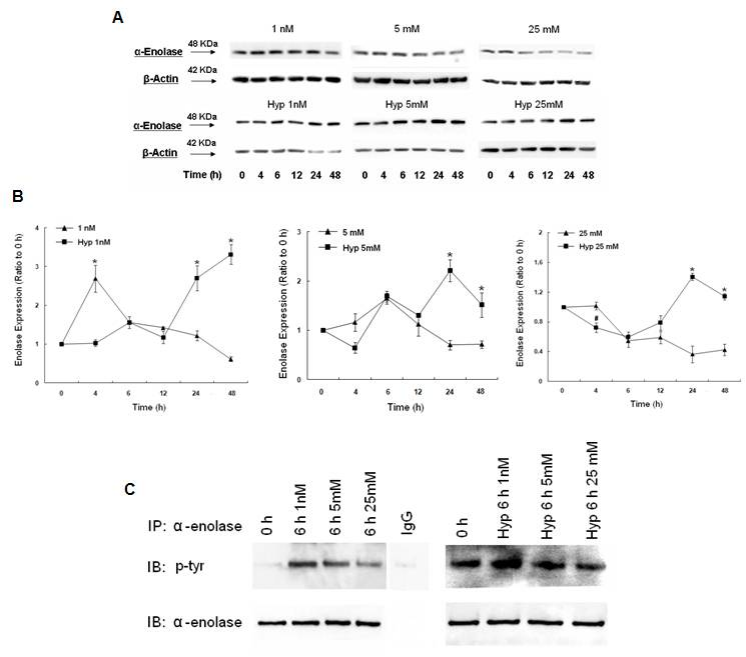
**Changes in α-enolase protein expression after hypoxia in response to 1 nM, 5 mM, or 25 mM glucose**. A) Western blots of α-enolase and β-actin (loading control) from total cell lysates. B) Histogram of α-enolase expression after 4, 6, 12, 24, or 48 h of hypoxia. Bars represent mean ± SEM expressed as a ratio to 0 h from 3 separate experiments. * denotes a significant (p < 0.05) increase in the level of expression.. Note greater induction of α-enolase expression after 48 h of hypoxia at all glucose concetrations compared to normoxia, especially with 1 nM glucose. Normoxia data was previously published [26]. C) Tyrosine phosphorylation of α-enolase after 6 h of hypoxia or normoxia with 1 nM, 5 mM, or 25 mM glucose. Total lysates were immunoprecipitated with α-enolase and immunoblotted with phosphotyrosine. Efficiency and specificity of the immunoprecipitations were verified by equal immunoprecipitation of α-enolase and an absence of α-enolase in samples immunoprecipitated with goat IgG. Note increased tyrosine phosphorylation of α-enolase in response to 1 mM or 5 mM glucose with hypoxia or normoxia.

These changes in α-enolase transcript levels in response to hypoxia were reflected in increased α-enolase protein expression (Fig. 4A&B). In hypoxic cells grown under low glucose conditions, there was a gradual increase in α-enolase expression, culminating to a 6-fold increase at 48 h compared to normoxia, which paralleled the rise in α-enolase mRNA. In the normoglycemic (5 mM) cells, the increase in α-enolase protein concentration was more modest, reaching a 3-fold increase at 24 h compared to normoxia and decreasing to 2-fold above normoxia at 48 h. In hypoxic cells grown in 25 mM glucose, α-enolase expression was initially decreased at 4 h compared to normoxia, however, increased 4-fold compared to normoxia at 24 h and decreased slightly to 3-fold at 48 h. Thus, hypoxia induced a greater increase in α-enolase protein expression by MCF-7 cells at lower concentrations of glucose (1 nM and 5 mM), although the level of α-enolase mRNA was increased at all glucose concentrations tested. This pattern is quite different from normoxic cells grown in high glucose, which showed an immediate rise in α-enolase protein expression and then a sharp decrease from 6-48 h [26].

### Changes in tyrosine phosphorylation of α-enolase with Hypoxia

Interestingly, α-enolase is one of only three glycolytic enzymes which can undergo tyrosine phosphorylation in transformed cells [27] and may be one mechanism by which transformed cells respond quickly to changes in ATP [28]. We decided to look at 6 h of normoxia and hypoxia since α-enolase expression was elevated in the 1 nM and 5 mM groups in the absence of an increase in α-enolase mRNA. After 6 h of hypoxia, there was a marked increase in tyrosine phosphorylation of α-enolase in the low glucose group compared to the 0 h normoxia control (Fig 4C). In the 5 mM glucose group, tyrosine phosphorylation was also elevated compared to the 0 h normoxia control, however the increase in phosphorylation was not as great as in the low glucose group. After 6 h of hypoxia in response to 25 mM glucose, tyrosine phosphorylation of α-enolase was not increased. Therefore, the increase in tyrosine phosphorylation of α-enolase at 6 h in response to hypoxia was dependent on glucose concentration, since increased tyrosine phosphorylation of α-enolase was also measured under normoxic conditions. These results suggest that the early hypoxia-induced changes in α-enolase protein expression may be mediated by post-translational modifications, which are influenced by glucose concentration.

### Effect of Hypoxia on MBP-1 Expression

**Figure 5:**
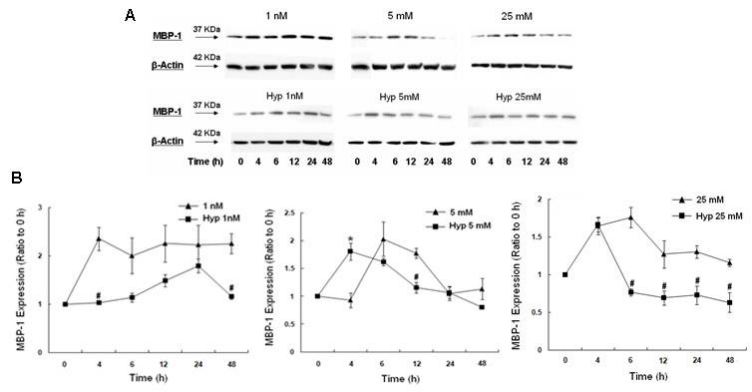
**Effect of hypoxia on MBP-1 protein expression in response to 1 nM, 5 mM, or 25 mM glucose**. A) Western blots of MBP-1 and β-actin (loading control) from total cell lysates. B) Histogram of MBP-1 expression after 4, 6, 12, 24, or 48 h of hypoxia. Bars represent mean ± SEM expressed as a ratio to 0 h from 3 separate experiments. * denotes a significant (p < 0.05) increase in expression and # denotes a significant decrease compared to normoxia. Note the brief, early increase in MBP-1 expression in cells exposed to hypoxia and 5 mM or 25 mM glucose and the brief, late increase in MBP-1 expression with hypoxia and 1 nM glucose. Overall, hypoxia attenuated MBP-1 expression at all glucose concentrations compared to normoxia. Normoxia data was previously published [26].

Since α-enolase and MBP-1 are products of the same mRNA, it is important to compare the effect of hypoxia on both translation products. Indeed, there was an interesting dichotomy in the effect of hypoxia on MBP-1 expression. Hypoxic cells grown in low glucose demonstrated a small, gradual increase in MBP-1 levels, which culminated to a peak at 24 h (Fig 5). By 48 h, MBP-1 levels had fallen to expression levels comparable to baseline. This is in marked contrast to normoxia, which showed a robust increase in MBP-1 expression that was maintained through 48 h. On the other hand, hypoxic cells grown in normal or high glucose, demonstrated an initial increase in MBP-1 expression, with a rapid, subsequent decline to baseline levels. Therefore, hypoxia attenuated MBP-1 expression at all glucose concentrations, however, the higher the glucose concentration, the faster the rate of decline in MBP-1.

### Hypoxia induces preferential translation of α-enolase

It is important to compare changes in the α-enolase/MBP-1 ratio in order to determine whether hypoxia may differentially affect the translation of either α-enolase or MBP-1. With hypoxic cells grown in low glucose, equal amounts of MBP-1 and α-enolase were translated initially, however, the delayed rise in MBP-1 expression at 12 h decreased the ratio to 0.79 at 12 h. After 24 and 48 h, α-enolase was preferentially translated over MBP-1 culminating to a ratio of 2.87 at which point hypoxic cells grown in low glucose start to proliferate (Fig 1). In hypoxic cells grown under both physiologic and high glucose conditions, there was an almost immediate decrease in the α-enolase/MBP-1 ratio (0.36 and 0.44 at 4 h, respectively) reflecting, primarily, a significant increase in MBP-1 expression. This was followed by a steady increase in the ratio at 48 h to 1.89 and 1.81 respectively, reflecting increased levels of enolase and a relative decrease in MBP-1 concentration. The immediate spike in MBP-1 expression and later increase in α-enolase in hypoxic cells grown in normal and high glucose concentrations is consistent with the delay in cellular proliferation (Fig 1). Under normoxic conditions at all glucose concentrations, the α-enolase/MBP-1 ratio is consistently less than 1.0 indicating that, MBP-1 is preferably translated over α-enolase [26]. These results indicate that hypoxia induces preferential translation of α-enolase at all glucose concentrations at 24 and 48 h, however the greatest increase in the α-enolase/MBP-1 ratio occurred with 1 nM glucose.

### Effect of Hypoxia on c-myc expression

**Figure 6:**
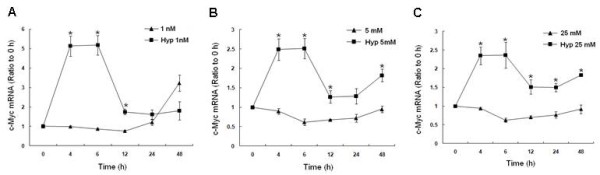
**Hypoxia-induced changes in c-myc mRNA in response to 1 nM, 5 mM, or 25 mM glucose at 4, 6, 12, 24, or 48 h by RT-PCR**. All measurements were standardized with β-actin (loading control) and expressed as a ratio to the 0 h (expression ratio). Bars represent mean ± SEM from 3 separate experiments. * denotes a significant (p < 0.05) increase in mRNA compared to normoxia. Note the early robust increase in c-myc transcripts in response to hypoxia that was greatest with 1 nM glucose. No early increase in c-myc mRNA occurred with normoxia. Normoxia data was previously published [26].

The alternative α-enolase translation product, MBP-1, negatively regulates c-myc transcription. In order to characterize the physiologic effect of hypoxia induced MBP-1, corresponding changes in c-myc mRNA were assessed by RT-PCR. There was a 5-fold rise in c-myc mRNA in hypoxic cells grown in 1 nM glucose as early as 4 h compared to normoxia (Fig 6). However, c-myc transcript levels decreased to 2-fold above baseline, nearly the same as normoxic levels from 24-48 h. The pattern of the c-myc response was the same but less prominent in cells grown in physiologic and high glucose, reaching 2.5-fold above normoxia. These results suggest that hypoxia stimulates an early (4-6) increase in c-myc mRNA at all glucose levels tested, The level of induction was about 2-fold greater in the low glucose group compared to normal and high glucose.

**Figure 7:**
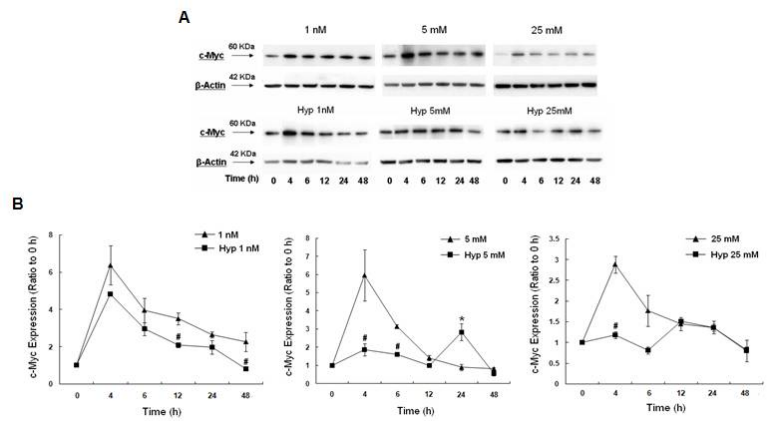
**Changes in c-myc protein expression after hypoxia in response to 1 nM, 5 mM, or 25 mM glucose**. A) Western blots of c-myc and β-actin (loading control) from total cell lysates. B) Histogram of c-myc expression after 4, 6, 12, 24, or 48 h of hypoxia. Bars represent mean ± SEM expressed as a ratio to the 0 h from 3 separate experiments. * denotes a significant (p < 0.05) increase in expression and # denotes a significant decrease compared to normoxia. Note attenuation of the early increase in c-myc protein in response to 5 mM or 25 mM glucose with hypoxia. Normoxia data was previously published [26].

To determine whether changes in c-myc mRNA levels paralleled changes in c-myc protein, c-myc expression was assessed by Western blot analysis. Hypoxic cells grown in low glucose demonstrated a robust increase in c-myc protein expression after 4 h, which gradually decreased to baseline levels by 48 h, a pattern very similar to normoxia (Fig 7). With hypoxic cells grown in 5 mM glucose, the initial c-myc response was less prominent and markedly decreased compared to normoxia. When hypoxic cells were grown in 25 mM glucose, the early increase in c-myc expression was attenuated and not different from baseline. These results indicate that c-myc protein expression is induced early in response to hypoxia with lower levels of glucose (1 nM and 5 mM) in parallel with increased c-myc mRNA. However, hypoxia with 25 mM glucose prevented the early increase in c-myc expression. Both 5 mM and 25 mM glucose with hypoxia induced a later increase (24 h) in c-myc expression independent of an increase in c-myc transcripts.

### Hypoxia increases the production of ROS

**Figure 8:**
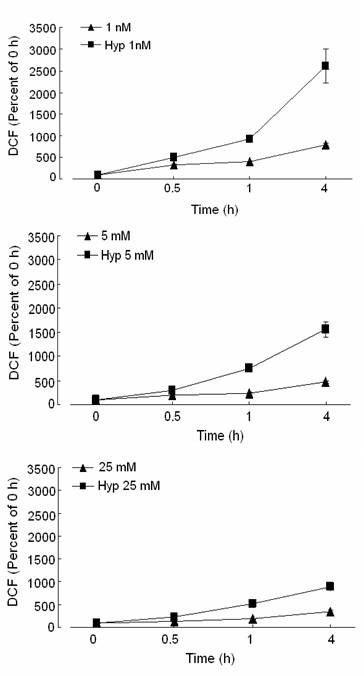
**Effect of hypoxia on ROS production in response to 1 nM, 5 mM, or 25 mM glucose**. ROS production was measured in media and in cells after 0.5, 1, or 4 h of hypoxia (square) or normoxia (triangle) by the conversion of 2',7'-dichlorofluorescin diacetate (DCFH-DA) to 2',7'-dichlorofluorescein (DCF) and expressed as a percent of the 0 h. Note increased ROS production with hypoxia compared to normoxia, which was greatest at 4 h with 1 nM glucose.

To determine whether the early increase in c-myc mRNA was associated with an increase in oxidative stress, we assessed changes in intracellular ROS. The ROS levels were assessed with DCFH-DA, which forms the fluorescent compound DCF when oxidized. Hypoxic cells demonstrated an early increase in intracellular ROS generation at all glucose concentrations, compared to normoxic cells (Fig 8). After 4 h of hypoxia, intracellular ROS production increased by more than 25-fold in cells grown in low glucose, 15-fold in physiologic glucose, and 8-fold in high glucose. At all times, the level of ROS was great in hypoxic cells compared to cells grown under normoxia. Clearly, hypoxia increased intracellular ROS production, particularly in cells grown in low glucose. Higher concentrations of glucose attenuated the increase in ROS production under hypoxia and normoxia.

### Hypoxia reduces MBP-1 binding to the c-Myc P_2 _Promoter

**Figure 9:**
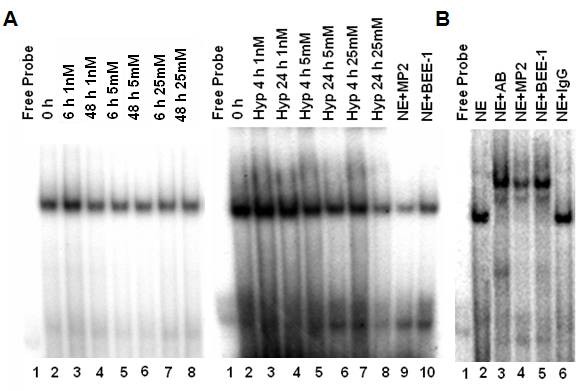
**Influence of hypoxia on P_2 _promoter binding by c-myc promoter binding protein in response to 1 nM, 5 mM, or 25 mM glucose as determined by electrophoretic mobility shift assay**. A) A 50 base pair c-myc P_2 _promoter was ^32^P-labeled and incubated with nuclear extract (NE). Cold competitor MP2 (150 nM) (Lane 9) or cold nonspecific oligonucleotide BEE-1 (150 nM) (Lane 10) was added prior to addition of the probe. Note that MBP-1 binding to the probe with 1 nM glucose was similar to the 0 h after 4 and 24 h of hypoxia. This is in contrast to normoxia data which showed an early increase in binding compared to baseline. Binding was greatly reduced in the 5 mM and 25 mM groups after 4 and 24 h of hypoxia compared to normoxia. B) An α-enolase antibody was added to a reaction containing the nuclear extract and ^32^P-labeled P_2 _promoter (lane 3) causing a supershift of the DNA-protein complex. This supershift was competed off with MP2 cold competitor (lane 4), but not with the cold nonspecific competitor BEE-1 (lane 5). No supershift occurred with addition of rabbit IgG to the reaction in place of antibody (lane 6). Normoxia figure was adapted and previously published [26].

MBP-1 has previously been shown to bind to a DNA sequence containing the TATA box of the c-myc P_2 _promoter and downregulate its transcription. Since hypoxia caused an early induction in c-myc transcripts and MBP-1 is a negative regulator of c-myc transcription, we were interested in whether a change in MBP-1 binding to the c-myc P_2 _promoter occurred. This question was addressed by EMSA using a 50 bp ^32^P-labeled oligonucleotide relative to the P_2 _start site on the c-myc promoter (Fig. 9A). A DNA-protein complex was visualized by autoradiography, and its specificity was confirmed when the complex was disrupted with 150 nM excess of cold MBP-1 competitor (MP2), but not with 150 nM excess of the cold non-specific competitor BEE-1. After 4 h of hypoxia with 1 nM glucose, there was little difference in P_2 _promoter binding compared to the 0 h normoxic control. However, the addition of hypoxia prevented the increase in promoter binding previously seen with normoxia [26]. After 24 h of hypoxia with low glucose, MBP-1 binding to the P_2 _promoter decreased slightly, although MBP-1 expression was elevated. On the other hand, although MBP-1 protein expression was elevated after 4 h of hypoxia with 5 mM and 25 mM glucose, binding to the P_2 _promoter was decreased compared to 0 h, similar to normoxia. However, after 24 h of hypoxia, MBP-1 binding to the P_2 _promoter drastically decreased and the greatest reduction in binding was measured with 25 mM glucose. This decrease in binding was greater with hypoxia compared to normoxia Therefore, hypoxia markedly decreased binding of MBP-1 to the c-myc P_2 _promoter coincident with the increase in c-myc transcripts. An α-enolase polyclonal antibody was able to bind and supershift the DNA-protein complex and could be effectively competed with excess MP2 but not BEE-1, further demonstrating specificity of the shifted complex (Fig. 9B).

### Effect of glucose concentration and hypoxia on Glut-1 mRNA expression

**Figure 10:**
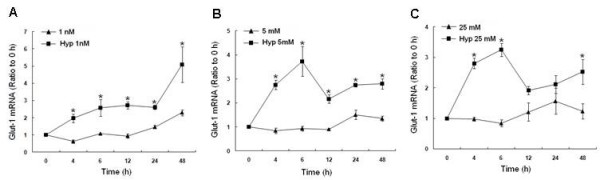
**Hypoxia-induced changes in glut-1 mRNA in response to 1 nM, 5 mM, or 25 mM glucose at 4, 6, 12, 24, or 48 h by RT-PCR**. All measurements were standardized with β-actin (loading control) and expressed as a ratio to the 0 h (expression ratio). Bars represent mean ± SEM from 3 separate experiments. * denotes a significant (p < 0.05) increase in mRNA compared to normoxia. Note early stimulation of glut-1 transcripts in response to hypoxia and the robust increase in glut-1 mRNA at 48 h with 1 nM glucose. No early increase in glut-1 transcripts occurred with normoxia. Normoxia data was previously published [26].

Glut-1 is a glucose transporter markedly induced by hypoglycemia [26]. It is also an ideal candidate gene which may be involved in the cellular response to hypoxia and altered glucose concentrations. Changes in glut-1 mRNA were assessed with RT-PCR (Fig. 10). In hypoxic cells grown in 1 nM glucose, there was an almost immediate 2-fold increase in glut-1 transcription that reached 3-fold greater than normoxia by 48 h. In hypoxic cells grown in physiologic or high glucose, there was also an immediate increase in glut-1 mRNA reaching about 2-fold greater than normoxia, however the overall response by 48 h was less dramatic than that seen with hypoxia and low glucose. Therefore, hypoxia stimulated an increase in glut-1 transcripts at all glucose concentrations compared normoxia. However, cells grown under hypoxia in low glucose induced the greatest increase in glut-1 transcription after 48 h.

## Discussion

Even in the presence of adequate oxygen, transformed cells metabolize the majority of the glucose they take up through glycolysis [29]. This results in an "addiction" to glucose as the source of ATP production, while available lipids and amino acids are redirected for use in anabolic synthesis [30]. C-Myc regulates many genes that are responsible for these metabolic differences between normal and malignant cells. However, the exact molecular mechanism by which the "aerobic glycolysis" of cancer cells (Warburg effect) offers a selective growth advantage for tumors remains unclear.

We have previously reported that the alternative translation product of the glycolytic enzyme α-enolase, MBP-1, functions as a tumor suppressor because of its ability to bind to the c-myc promoter and downregulate c-myc mRNA and inhibit cellular proliferation [31]. Recently, we determined that both expression and function of MBP-1 are regulated by alterations in exogenous glucose concentrations and correspond to changes in cell proliferation and lactate production [26]. However, the differential translation of α-enolase and MBP-1 and its relation to the control of cell growth and metabolism under hypoxia have not been characterized.

The c-myc oncogene plays an important role during the cellular response to hypoxia to help achieve oxygen homeostasis which is required for cell survival, promoting glucose transport and enhanced glycolysis [32]. The α-enolase gene, which encodes a glycolytic enzyme (α-enolase) whose expression is stimulated by c-myc, and a DNA binding protein (MBP-1), which binds the c-myc promoter and downregulates c-myc expression, is in a unique position to integrate the cellular response to hypoxia. Our data support the hypothesis that preferential translation of α-enolase and inhibition of MBP-1 function play a role in the adaptation of MCF-7 cells to low oxygen, particularly under low glucose conditions. This adaptation is likely to be critical for the progression and metastasis of tumor cells.

The role of MBP-1 in the hypoxic response is evidenced in several ways. Hypoxic MCF-7 cells have a normal growth rate, except in low glucose, suggesting that they have developed molecular adaptations to hypoxia. This suggests that in these transformed cells, enhanced glucose utilization allows proliferation, even in hypoxic conditions. This is reflected in the increased lactate production by the hypoxic cells in normal and high glucose concentrations compared to normoxia. On the other hand, hypoxic cells grown in low glucose demonstrated both a decreased growth and rate of lactate production.

It has been previously reported that expression of α-enolase is stimulated by hypoxic conditions and that this increase is mediated by both c-myc and Hif-1α since the Hif-1α binding site in the α-enolase promoter (ChoRE, carbohydrate response element) overlaps with the c-myc E-box binding site [33]. As a glycolytic pathway enzyme, this stimulation serves to increase the overall level of glycolysis in response to low oxygen availability. However, in contrast to the other glycolytic pathway enzymes, the α-enolase gene also encodes a tumor suppressor gene, MBP-1, which downregulates c-myc expression [20]. Our data confirms that hypoxia stimulates α-enolase mRNA levels at all glucose concentrations compared to normoxia. This increase was more pronounced in hypoxic cells grown in low (6-fold) and high (3-fold) glucose concentrations. The increase in α-enolase mRNA in response to hypoxia at all glucose concentrations resulted in a corresponding increase in α-enolase protein and the α-enolase/MBP-1 ratio compared to normoxia. Since lactate production increased in the medium of the 5 mM and 25 mM hypoxia groups concurrently with the rise in α-enolase mRNA and protein expression suggests a possible contribution of α-enolase to the hyperglycolytic rate of cancer cells.

MCF-7 cells grown in normoxic conditions with low glucose demonstrated an early (4 h) increase in MBP-1 expression [26] which was accompanied by a striking increase in MBP-1 binding activity to the c-myc promoter by EMSA analysis. On the other hand, hypoxic cells had an attenuated MBP-1 response at all glucose concentrations compared to normoxia. In accordance with the attenuated changes in MBP-1 protein expression in hypoxic cells, binding of MBP-1 to the c-myc P_2 _promoter was unchanged in relation to the 0 h control. In contrast, hypoxic cells grown in physiologic or high glucose demonstrated a short-lived increase in MBP-1 expression at 4-6 h. This was followed by a return to baseline levels and a marked decrease of MBP-1 binding to the c-myc P_2 _promoter, corresponding to a steady increase in proliferation through 48 h. MCF-7 cells exposed to physiological or high glucose with hypoxia are rapidly proliferating and metabolizing glucose. An early increase in MBP-1 expression may serve to initially limit cell proliferation to allow for upregulation of alternative metabolic pathways leading to production of lactate. This strong inverse correlation between MBP-1 expression levels and rate of cellular proliferation is consistent with previous reports that it functions as a tumor suppressor in breast and prostate cancer cells [24,34].

Although the absolute concentration of α-enolase protein was higher than MBP-1 under all conditions, the relative levels of translation of the two enolase gene products were quite different. Initially, under conditions of normal and high glucose, in the presence of increased α-enolase mRNA, the concentration of MBP-1 was increased over α-enolase, suggesting preferential translation of MBP-1. On the other hand, initially at low glucose concentrations, MCF-7 cells had similar increases in α-enolase and MBP-1, suggesting that these cells, under considerable metabolic stress increased the level of both gene products. However, at 48 h, α-enolase was preferentially translated above the levels measured for normal and high glucose.

In general, hypoxic cells demonstrated changes in c-myc mRNA which were consistent with their levels of MBP-1 expression and c-myc promoter binding. For example, in hypoxic cells grown in low glucose, the failure of these cells to generate an early increase in MBP-1 allowed the corresponding robust 5-fold increase in c-myc expression. This is consistent with studies which have shown regulation of c-myc mRNA by MBP-1 expression and binding to the c-myc P_2 _promoter in normoxic cells [26]. These results are consistent with the role of c-myc as an "early response" gene whose transcript is stimulated immediately with hypoxia (2-6 fold depending on the tissue type) [35].

On the other hand, c-myc protein expression also paralleled c-myc transcript levels in cells grown in 5 mM glucose and hypoxia. The failure of these cells to mount an early MBP-1 response allowed a 2-fold increase in c-myc mRNA, which corresponded to a 2-fold increase in protein expression and resulted in cellular proliferation. Together, these results suggest that hypoxia in the presence of low glucose attenuates translation of MBP-1 and disrupts MBP-1-mediated regulation of c-myc transcription by inhibiting binding of MBP-1 to the c-myc P_2 _promoter. Since c-myc is deregulated in most cancers [36], inhibition of MBP-1 function may be one mechanism which perpetuates c-myc overexpression at the mRNA and protein level. Inhibition of MBP-1 function may also decrease the susceptibility of cells to apoptosis as shown with human fibroblasts [37], and carcinoma [38]. Therefore, loss of MBP-1 function may be an important adaptation which allows transformed cells to survive under limited glucose and oxygen availability.

Interestingly, α-enolase is one of only three glycolytic enzymes which undergo tyrosine phosphorylation in transformed cells, however, the significance of this phosphorylation is unknown [39]. In this study, increased tyrosine phosphorylation of α-enolase was observed in hypoxic cells grown in low and normal glucose levels, corresponding to the early rise in α-enolase protein expression. This may be a mechanism by which transformed cells respond quickly to changes in ATP [27], although the significance of α-enolase tyrosine phosphorylation remains unclear. Several reports have shown direct correlations between increased expression of enolase both at the RNA and protein level and the progression of tumors [40]. These results suggest that hypoxia induces preferential translation of α-enolase over MBP-1 and may be important for early tumor cell adaptation to oxidative stress.

Several studies have shown that hypoxia increases the production of ROS and depletes ATP [41], which may increase c-myc transcription "early c-myc response" [42]. In the current study, hypoxia drastically increased intracellular ROS at all glucose concentrations after 4 h compared to normoxic conditions, corresponding to the robust increase in c-myc mRNA. However, transcript levels of c-myc mRNA after 4 h of hypoxia were particularly elevated for the 1 nM glucose group and corresponded with greater ROS production compared to the 5 mM and 25 mM glucose groups. Since glucose deprivation results in mitochondrial dysfunction and enhances cellular sensitivity to oxidative stress, robust quantities of ROS are produced [43,44] resulting in higher levels of c-myc mRNA. Previous studies show that c-myc is markedly upregulated in response to superoxide dismutase deficiency suggesting that early upregulation of c-myc may play a role in helping cells overcome oxidative stress [45]. In our study, when cells were exposed to 25 mM glucose in a hypoxic environment, ROS production was markedly attenuated. Since high glucose may prevent mitochondrial dysfunction and be protective against hypoxic injury [46], ROS levels may be correspondingly reduced resulting in lower c-myc transcript levels. Our results suggest that induction of c-myc is dependent on the quantity of ROS produced and the susceptibility of the cells to injury by ROS.

The stimulation of glycolysis in hypoxic cells is driven, in part, by an increased rate of glucose transport [47]. Consistent with their concordant actions in other aspects of glucose metabolism, the glucose transporter, glut-1, is transactivated by both c-myc and Hif-1α [15]. Its overexpression correlates with poor prognosis and tumor aggressiveness in cancer patients [48]. Our results show an early 2-fold increase in glut-1 mRNA in response to hypoxia. This increase was persistent, with increased expression as long as the cells remained hypoxic. Interestingly, hypoxic cells grown in low glucose demonstrated a 3-fold increase in glut-1 mRNA after 48 h of hypoxia compared to normoxia, paralleling the large increase in α-enolase mRNA. At all glucose concentrations, transcript levels of glut-1 after hypoxia were significantly greater than those of normoxic cells [26].

## Conclusions

In summary, the present study demonstrates the important role of α-enolase and MBP-1 in the changes in cell proliferation and glycolysis induced by hypoxia. Our results demonstrate that exposure to hypoxia attenuates translation of MBP-1 and disrupts MBP-1-mediated regulation of c-myc transcription by inhibiting binding of MBP-1 to the c-myc P_2 _promoter, particularly in cells grown in low glucose concentrations. This allows for immediate upregulation of c-myc, which may stimulate aerobic glycolysis and be instrumental in helping MCF-7 cells overcome oxidative stress. Preferential translation of α-enolase and its post-translational modification may play a critical role in the adaptation of MCF-7 cells to low oxygen and increased oxidative stress, particularly under low glucose concentrations (Table 1). This important "feedback" mechanism may help transformed cells to escape the apoptotic cascade, allowing for survival during limited glucose and oxygen availability. The important mechanisms modulating preferential translation of α-enolase or MBP-1 remain to be investigated.

**Table 1:** Summary of hypoxia-induced changes compared to normoxia

	Low Glucose	Normal Glucose	High Glucose
**Growth Rate**	↔	↑	↑
**Lactate**	↔	↑↑↑	↑↑↑
**Enolase mRNA**	↑↑	↑	↑↑↑
**Enolase Protein**	↑↑↑	↑↑	↑
**MBP-1 Protein**	↓↓	↓	↓↓
**c-Myc mRNA**	↑↑↑	↑↑	↑↑
**c-Myc Protein**	↓	↓↓	↓↓
**ROS**	↑↑↑	↑↑	↑
**Glut-1**	↑↑↑	↑↑	↑↑

* (↑) denotes an increase, **(↓) **denotes a decrease, and **(↔) **denotes no change compared to normoxia

## Methods

### Cell Culture

MCF-7 cells were purchased from the American Type Culture Collection (ATCC, USA) and maintained in 5% CO_2 _at 37°C in DMEM containing 25 mM glucose and 4 mM glutamine supplemented with 10% charcoal stripped FBS and 100 U penicillin/streptomysin. For experiments, cells were plated and allowed to settle overnight. The next day, cells were washed with glucose-free DMEM medium and incubated with DMEM containing 1 nM (low), 5 mM (physiologic), or 25 mM (high) glucose supplemented with 10% dialyzed FBS (Invitrogen) and 100 U penicillin/streptomysin at 37°C in a humidified gas-tight sealed chamber (Billups-Rothenburg, Del Mar, CA) gassed with 1% O_2_, 5% CO_2_, and balance N_2_. Cells were harvested at 0 h (before changing the medium, normoxia control) and at various times and used for subsequent experimental analysis.

### Cell Proliferation Assay

Cells were plated in a 6-well plate at 40% confluence. Cell counts and viability were determined by trypan blue exclusion counting on a hemocytometer at various time intervals.

### Cell Cycle Analysis

Cells were plated in a 60 mm plate at a density of 5 × 10^5 ^cells/plate. After 48 h of incubation under hypoxia or normoxia, the cells were collected in cold PBS, prepared and stained with the CycleTEST™ Plus DNA Reagent kit (BD Biosciences), which stains isolated nuclei with propidium iodide, and analyzed with a flow cytometer.

### Lactate Assay

Cells were plated in a 6-well plate at a density of 7.5 × 10^4 ^cells/well in 2 ml of medium. The next day, the medium was suctioned off and cells were rinsed with glucose free/phenol-red free DMEM and incubated in 1 nM, 5 mM, or 25 mM glucose medium (phenol-red free) at 37°C in a humidified chamber gassed with 1% O_2 _and 5% CO_2_. Medium was collected and lactate concentrations were determined by a previously described method [26]. This method monitors the NADH product at 340 nm after the NAD-linked conversion of lactate to pyruvate by lactate dehydrogenase with hydrazine trapping of pyruvate to ensure the reaction goes to completion. After 30 min of incubation at 25°C, absorbance was read at 340 nm and compared to a linear lactate standard curve (2-100 μg/ml). Medium blanks showed negligible absorbance.

### Analysis of ROS Generation

Generation of ROS in MCF-7 cells were assessed by using the probe 2',7'-dichlorofluorescin diacetate (DCFH-DA) (Sigma Chemical), a lipid-permeable non-fluorescent compound that when oxidized by intracellular reactive oxygen species (ROS), forms the fluorescent compound 2', 7'-dichlorofluorescein (DCF). MCF-7 cells were plated in a 6-well plate at a density of 7.5 × 10^4 ^cells/well in 2 mls of medium. The next day, the medium was aspirated and cells were rinsed with glucose free/phenol-red free DMEM and incubated in 1 nM, 5 mM, or 25 mM glucose medium (phenol-red free) containing 10 μM DCFH-DA (final concentration from 10 mM stock of DCFH-DA in DMSO) at 37°C in a humidified chamber gassed with 1% O_2 _and 5% CO_2 _for 0.5, 1, or 4 h. At the designated time of analysis, the medium containing the DCFH-DA was removed and the cells were rinsed with serum-free, phenol-red free medium and lysed with reporter lysis buffer (Promega). The lysates and media were transferred to a black 96-well plate and the fluorescence intensity of DCF was read at 538 nm emission and 485 nm excitation. Due to leakage of DCF across the cell membrane into the medium, DCF fluorescence was measured from the medium and the cells and together represents a qualitative estimation of intracellular ROS formation [49]. Negative controls containing DMSO instead of DCFH-DA showed negligible fluorescence.

### Western Blotting for α-enolase, MBP-1, and c-Myc

Cells were plated in a 60 mm culture dish at a density of 5 × 10^5^/plate and subjected to the experimental protocol as previously mentioned. To prepare protein extracts, cells were collected from each of the three treatment groups and lysed with Mammalian Extraction Reagent (M-PER) (Pierce) containing protease (Complete Mini, Roche) and phosphatase inhibitors (PhosphoStop, Roche). Thirty micrograms of total cell lysate was separated by SDS-PAGE on a 4-15% gradient denaturing gel and electroblotted onto PVDF membranes. Gel transfer efficiency and equal loading of proteins was verified by Ponceau S staining of PVDF membranes. The membranes were blocked for 1 h with 5% nonfat milk in phosphate buffered saline with 0.05% Tween-20 (PBS-T) and incubated overnight at 4 C with an α-enolase (C-19) or c-myc primary antibody (9E10) (Santa Cruz Biotechnology). After washing with PBS-T, the membranes were incubated with a horseradish peroxidase (HRP)-conjugated secondary antibody. Proteins were visualized using standard chemiluminescence (ECL) methods (GE Healthcare). Equal loading of proteins was verified by probing the membrane with a monoclonal β-actin primary antibody (Sigma Chemical). All films were scanned with an optical scanner (Epson Expression 1680) and quantified by measuring the density of each band using UNSCAN-IT software (Silk Scientific, Inc; Orem, UT). To correct for possible unequal loading, each band's density was normalized to its β-actin density. To allow for multiple comparisons between gels, each sample was compared to its respective 0 h that was run on the same gel. Calculation of the ratio of α-enolase to MBP-1 was calculated by dividing the averages of each protein.

### Tyrosine Phosphorylation of α-enolase

To evaluate tyrosine phosphorylation of α-enolase, 500 μg of total protein was immunoprecipitated with 5 μg of an anti-enolase goat polyclonal antibody (C-19, Santa Cruz Biotechnology). The protein-antibody-bead complex was washed three times with a buffer containing 1.0 M Tris pH 7.5, 1 M NaCl, 0.5 M EDTA, 1% NP-40, and protease/phosphatase inhibitors. A control reaction was done in which the same amount of IgG (goat) was added in place of anti-enolase antibody. The beads were resuspended in Lammelli loading buffer and heated at 95°C for 5 min and centrifuged for 2 min. The resulting supernatants were separated by SDS-PAGE and transferred to PVDF membranes as described above. For detection of tyrosine phosphorylation of α-enolase, the membranes were blocked for 3 h in 5% BSA in PBS-T and incubated with a monoclonal anti-phosphotyrosine primary antibody (PY-20, Santa Cruz Biotechnology) overnight at 4°C. The membranes were washed and developed as described above. Verification of equal immunoprecipitation of α-enolase was achieved by stripping the membrane and reprobing for total α-enolase using an anti-enolase rabbit polyclonal antibody (H-300, Santa Cruz Biotechnology). Specificity of the immunoprecipitations was demonstrated by the absence of α-enolase in samples immunoprecipitated with goat IgG.

### RT-PCR

Total RNA was extracted from MCF-7 cells using TriZol (Invitrogen) reagent according to manufacturer's instructions. RNA purity was determined by A260/A280 ratio and quantified by A260. Preparation of cDNA and forward and reverse primers for α-enolase, c-myc, β-actin, and glut-1 were as previously described [26]. All primers were designed using Primer Express Software (Applied Biosystems). RT-PCR was performed for a uniform amount of cDNA using the Fast 7500 System (Applied Biosystems). Reactions were diluted 1:2 with SYBR Green I Master Mix (Applied Biosystems) and amplification by PCR was as follows: 1 repetition at 50°C for 2 min, 1 repetition at 95°C for 10 min, and 40 repetitions of 95°C for 15 sec and 60°C for 1 min representing the melting, primer annealing, and primer extension phases of the reaction respectively. A no template control reaction was run for each gene to control for DNA contamination of RNA extracts. Following amplification, a dissociation curve was performed to provide evidence for a single reaction product. Message of α-enolase, c-myc, and glut-1 was compared to the 0 h control to calculate an expression ratio.

### Electrophoretic Mobility Shift Assay

To assemble the P_2 _promoter of c-myc, a 50 bp oligonucleotide (-52 to +2 relative to the P_2 _start site on the c-myc promoter) was radiolabeled using [γ-^32^P]-dATP with T_4 _polynucleotide kinase (Gibco BRL). It was annealed to its complement by heating at 90 C for 10 min in 20 mM Tris-HCl (pH 7.4), 10 mM Mg Cl_2 _and slow cooling to room temperature to form a radiolabeled double stranded probe. Nuclear extracts were prepared as previously described [50] from samples taken after 4 or 24 h of hypoxia from 1 nM, 5 mM, or 25 mM glucose concentrations. The probe (50,000 cpm) was incubated with 5 μg of nuclear extract in 20 mM Tris-HCl (pH 7.4), 140 mM KCl, 2.5 mM MgCl_2_, 1 mM DTT, 8% v/v glycerol, and 0.2 mM PMSF for 30 min at 37 C. In some reactions, 150 nM of the unlabeled competitor MP2 5'-AGGGATCGCGCTGAGTATAAAAGCCGGTTTTCGGGG-3' containing the binding site for MBP-1 or the nonspecific competitor BEE-1 5'-AGCTGTTCTGAGTGGGG GAGGGGGCTGCGCCTGC-3', containing an unrelated consensus sequence, were added before adding the probe to demonstrate specificity. A supershift was performed as described, except that 5 μg of anti-α-enolase polyclonal antibody (Abcam) was added before addition of the probe for 30 min at 4°C. The protein-DNA binding reactions were separated by electrophoresis on a 5% nondenaturing polyacrylamide gel at room temperature in 1× Tris borate-EDTA. Complexes were visualized by autoradiography.

### Data Analysis

All values represent mean ± SEM. Differences between normoxic and hypoxic samples were determined by a non-paired t-test (2-tailed). A probability level of p < 0.05 was used to indicate statistical significance.

## Abbreviations

MBP-1: c-myc binding protein; MCF-7: human breast carcinoma cells; KDa: kilodalton; HIF-1: hypoxia inducible factor: DMEM: Dulbecco's modified Eagle's medium; FBS: fetal bovine serum; DCFH-DA: 2',7'-dichlorofluorescin diacetate; DCF: 2',7'-dichlorofluorescin; M-PER: Mammalian Extraction Reagent; PBS: phosphate buffered saline; RT-PCR: real time polymerase chain reaction; EMSA: electrophoretic mobility shift assay.

## Competing interests

The authors declare that they have no competing interests.

## Authors' contributions

KCS-participated in the design/coordination of the study, performed the experiments, interpreted data, drafted the manuscript. 

SDT-participated in the design/coordination of the study, provided technical assistance, interpreted data. 

DMM-conceived of the study, participated in the design/coordination of the study, interpreted data, helped draft/revise the manuscript. 

All authors read and approved the final manuscript.

## Pre-publication history

The pre-publication history for this paper can be accessed here:

http://www.biomedcentral.com/1471-2407/10/157/prepub
